# Drug-induced ER stress leads to induction of programmed cell death pathways of the malaria parasite

**DOI:** 10.1007/s00436-024-08281-3

**Published:** 2024-07-08

**Authors:** Sinem Unal, Umit Y. Kina, Mohd Kamil, Ahmed S. I. Aly, Bedia Palabiyik

**Affiliations:** 1 Aly Lab, Beykoz Institute of Life Sciences and Biotechnology, Bezmialem Vakif University, Istanbul, 34820 Turkey; 2 Department of Molecular Biology and Genetics, Institute of Graduate Studies in Sciences, Istanbul, University, 34134 Istanbul, Turkey; 3 Center for Global Infectious Disease Research, Seattle Children’s Research Institute, 307, Westlake Ave N, Seattle, WA USA; 4 School of Science and Engineering, Al Akhawayn University, 53000 Ifrane, Morocco; 5 Faculty of Science, Department of Molecular Biology and Genetics, Istanbul University, 34134 Istanbul, Turkey

**Keywords:** Malaria, *Plasmodium*, ER stress, Autophagy, Apoptosis

## Abstract

The rapid emergence of drug resistance against the mainstream antimalarial drugs has increased the need for development of novel drugs. Recent approaches have embarked on the repurposing of existing drugs to induce cell death via programmed cell death pathways. However, little is known about the ER stress response and programmed cell death pathways of the malaria parasite. In this study, we treated ex vivo *Plasmodium berghei* cultures with tunicamycin, 5-fluorouracil, and chloroquine as known stress inducer drugs to probe the transcriptional changes of autophagy and apoptosis-related genes (*Pb*ATG5, *Pb*ATG8, *Pb*ATG12, and *Pb*MCA2). Treatments with 5-fluorouracil and chloroquine resulted in the upregulation of all analyzed markers, yet the levels of *PbATG5* and *PbATG12* were dramatically higher in chloroquine-treated ex vivo cultures. In contrast, tunicamycin treatment resulted in the downregulation of both *PbATG8* and *PbATG12*, and upregulation of *PbMCA2*. Our results indicate that the malaria parasite responds to various ER stressors by inducing autophagy- and/or apoptosis-like pathways.

## Introduction

Malaria is a vector-borne infectious disease that causes almost a quarter billion cases and over half a million deaths each year (WMR 2023). The disease is caused by *Plasmodium* species protozoan parasites and spread by Anopheline mosquitoes. One of the main obstacles in the fight against malaria is the increasing drug resistance. Repurposing of existing drugs is a useful strategy to meet the urgent need for new drugs (Laudisi et al. 2019). Studies show that using severe and sustained ER stress to induce programmed cell death (PCD) pathways is an efficient method against cancer and parasitic protozoan infections (Peng et al. 2021; Zhang et al. 2019).

Endoplasmic reticulum (ER) homeostasis is maintained through well-conserved mechanisms in eukaryotes. It is a vital balance between protein load and capacity of ER folding machinery. When it is broken by either intracellular or extracellular stress conditions, unfolded protein response (UPR) is activated to reduce the ER load or to increase the folding capacity of ER (Walter and Ron 2011). There are three transmembrane proteins, responsible from three separate signaling pathways that mediates unfolded protein response in mammalian cells: activating transcription factor 6 (ATF6), inositol-requiring kinase/endoribonuclease 1 (IRE1), and protein kinase RNA-like ER kinase (PERK) (Ma and Hendershot 2001). During ER homeostasis, these proteins interact with a chaperone protein commonly known as glucose-regulated proteins 78 (GRP78) or immunoglobulin heavy chain binding protein (BiP) (Kozutsumi et al. 1988). When UPR is activated, BiP dissociates from the transmembrane proteins and binds to misfolded proteins. While dissociation of BiP activates IRE1 and ATF6 for transcriptional regulation of UPR by increasing secretion and chaperone capacity of ER, it also activates PERK for translational attenuation. Failure of these mechanisms to eliminate ER stress results in activation of various cell death pathways (Peng et al. 2021).

Malaria parasites have a secretome of at least 320 identified proteins (Hiller et al. 2004). Excessive amounts of protein trafficking are necessary for the establishment of infection in the host cell, which alone could overload ER to activate UPR. Malaria parasites harbor a highly reduced UPR, in which transcriptional regulators of the UPR are absent, but few homologues of UPR components such as BiP and PERK have been identified (Kaiser et al. 2016).

Although not fully understood, there are many studies reporting that *Plasmodium* parasites harbor apoptosis-like PCD pathways exhibiting common signs of apoptosis such as chromatin condensation and DNA fragmentation (Pollitt et al. 2010; Meslin et al. 2007; Le Chat et al. 2007; Al-Olayan et al. 2002; Picot et al. 1997). Malaria parasites do not possess classical caspase family genes; instead, they have homologues of metacaspase genes MCA1, MCA2, and MCA3. (Kumari et al. 2022; Kumar et al. 2019; Le Chat et al. 2007). MCA1 and MCA2 are thought to be involved in these unique cell death pathways (Timothy and Zininga 2023).

On the other hand, *Plasmodium* parasites contain homologs of almost half of the yeast autophagy proteins. Erythrocytic stages of the parasite were shown to undergo autophagy-like cell death mechanism in which autophagosome-like vacuoles were observed under certain drug stresses (Navale et al. 2014; Hain and Bosch 2013; Kamil et al. 2022).

In our study, we aimed to analyze the expression profiles of autophagy-related genes (*Pb*ATG5, *Pb*ATG8, and *Pb*ATG12) and a metacaspase gene, *Pb*MCA2, to probe the responses of the rodent malaria parasite *Plasmodium berghei* against well-known ER stress inducers. We reasoned that the ex vivo* Plasmodium berghei* culture would be a more appropriate model for measuring the effect of drug-induced ER stress for mimicking the mammalian infection due to the possibility of alterations in transcriptomics, virulence factors, and drug sensitivity of the extensively in vitro cultured, lab-adapted strains of *P. falciparum* (Brown and Guler 2020).

## Materials and methods

### Ethics statement

All animal experiments described here were performed in accordance with the Experimental Animals Ethical Committee of Bezmialem Vakif University, Istanbul, Turkey (no. 2020/235).

### Parasite cultures and drug treatment

Six to eight-week-old female CD1 mice were purchased from Bezmialem Vakif University, Experimental Animal Research Center. Donor mice were infected intraperitoneally with cryopreserved stocks of wild type-like eGFP expressing *P. berghei Pbp230p(-)* strain. This strain was recently shown to have similar growth characteristics as wild-type parasites (Kamil et al. 2024). Parasitemia of the donor mice was monitored by flow cytometry. Infected mice blood was collected by cardiac puncture when parasitemia reached 5%. Parasite life cycle stages were synchronized by culturing overnight in RPMI 1640 supplemented with 20% heat-inactivated FBS and 20 µg/mL gentamicin. Synchronized schizonts were collected by density gradient centrifugation. Briefly, 10 mL 60% (v/w) Accudenz solution was added gently into the bottom of a 35 mL parasite culture mix in a 50-mL centrifuge tube. Mix was carefully centrifuged at 200 *g* for 20 min with no brakes. Schizonts formed brown-gray ring in between two phases, and they were collected by using long, glass Pasteur pipettes. Synchronized schizonts were injected into naïve mice intravenously.

Mice infected with synchronized parasites were bled after 48 h by cardiac puncture, and collected blood was mixed with parasite culture medium. Culture was aliquoted into six-well plates to create ex vivo experiment groups (three wells per group). Parasitemia of cultures was monitored at the beginning and every 2.5 h at a dilution of 1:200 in PBS.

Non-lethal concentration of drugs was used that do not cause parasite death but induce cell stress (Unal 2022). The concentrations of drugs were 0.25 µg/mL chloroquine (CQ), 5 µg/mL tunicamycin (TN), and 2.5 µg/mL 5-fluorouracil (5-FU).

### RT-qPCR analysis

Three milliliters of cultures were collected at 5th hour, pelleted at 300 *g* for 5 min, and washed with 500 µL PBS. RNA isolation was performed by using RNeasy Kit (Qiagen), and cDNA synthesis was performed by using High-Capacity cDNA Reverse Transcription Kit (Applied Biosystems).

Synthesized cDNAs were used in qPCR analysis to probe ER stress-related genes (*PbBiP*), autophagy genes (*PbATG5*, *PbATG8*, and *PbATG12*), and a metacaspase gene (*PbMCA2*). 18S rRNA housekeeping gene was used as reference. Reactions were performed in BioRad CFx96 real-time PCR system by using iTaq SYBR Green Master Mix (BioRad) according to the manufacturer’s instructions. Primer sequences are given in Table 1.

**Table 1:** qPCR primers used in the study

Name	Sequence
ATG5_F	CTGCATGTGAATTTTCCCAC
ATG5_R	AAGGAGATTTTAATGGAATTTG
ATG8_F	CCCCTTTGAAAGTAGAGTTGCAG
ATG8_R	CGCTTTTTCACACACCACTGG
ATG12_F	TGGAAATGAAACACTAGTATCCA
ATG12_R	TCCAAATTGGGTTTAATCGTGTT
MCA2_F	AATGGACCCTTGTTCCATCC
MCA2_R	TTCCCCTTCATGAGCAACTC
BiP_F	CCCACTACTTTTGCACCTGA
BiP_R	TAAACCAGCAATTGCACCAG
18S_F	ACATGGCTTTGACGGGTAAC
18S_R	TGCTGCCTTCCTTAGATGTG

### Statistical analysis

Statistically significant differences between the median values of treatment groups were evaluated by the analysis of variance (ANOVA) method in all experiments. GraphPad Prism 9 software was used for all analyses. *p* values of 0.05 were considered statistically significant.

## Results

### Treatment with sub-lethal drug doses does not affect parasite growth

**Fig. 1:**
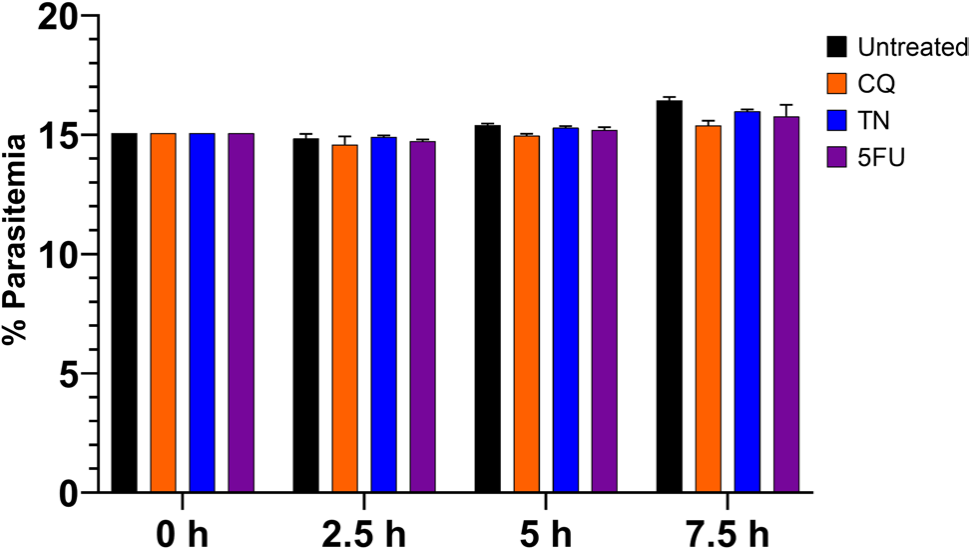
Drug treatments do not affect parasite growth. Parasite cultures were treated with 0.25 µg/mL CQ, 5 µg/mL TN, and 2.5 µg/mL 5-FU. Parasite growth was recorded every 2.5 h by flow cytometry. Experiments were performed in three replicates. Results were shown as percent parasitemia for each time point. Bars represent mean ± SD values for each treatment

Parasite cultures were treated with sub-lethal doses of CQ, TN, and 5-FU that do not cause death but generate stress response in the parasite (Unal 2022). Parasite survival was measured by flow cytometry every 2.5 h. As expected, applied drugs had no significant effect on parasite survival (Fig. 1).

### Drug treatments induce elevated levels of ER stress

**Fig. 2:**
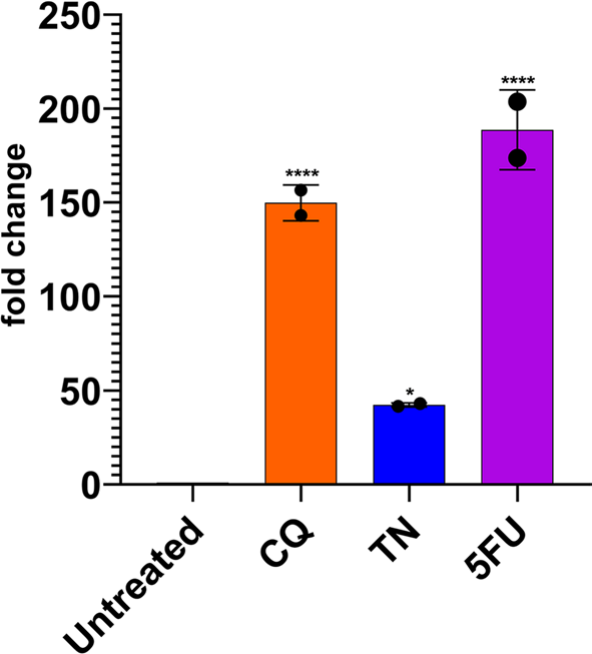
Drug treatments significantly upregulate *PbBiP* transcription. Parasite cultures were treated with 0.25 µg/mL CQ, 5 µg/mL TN, and 2.5 µg/mL 5-FU. Five hours later, total RNA was isolated for cDNA synthesis from 3 mL of culture wells. All qPCR reaction mixtures contained equal amount of cDNA from the treated or untreated cultures. Chloroquine (CQ), tunicamycin (TN), and 5-fluorouracil (5-FU) treatments caused ~ 150, ~ 40, and ~ 190-folds upregulation of *PbBiP*, respectively. Experiments were performed in three replicates. Results were shown as fold changes relative to the untreated control. Each bar shows mean ± SD values for each gene. *p < 0.05, and ****p < 0.0001

To evaluate whether the drugs induced ER stress, we checked the transcription profile of ER resident chaperone protein *GRP78 (BiP)* gene (Fig. 2). TN treatment upregulated *PbBiP* transcription ~ 40 folds, while CQ and 5-FU treatments upregulated ~ 150 and ~ 190 folds, respectively, indicating a strong ER stress response under all three drug treatments.

### Drug-induced ER stress induces upregulation of autophagy-related genes

**Fig. 3:**
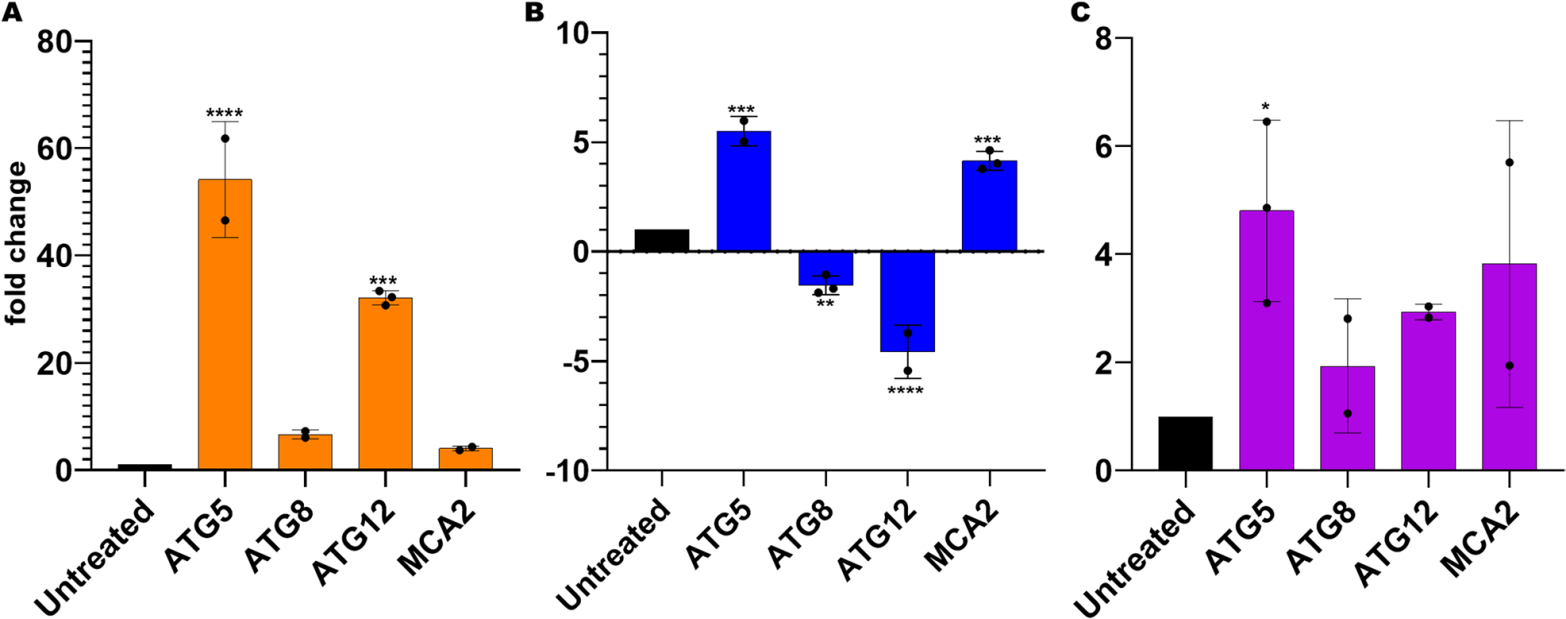
Drug-induced ER-stress alters the transcription levels of autophagy and apoptosis-related genes. Parasite cultures were treated with 0.25 µg/mL CQ, 5 µg/mL TN, and 2.5 µg/mL 5-FU. Five hours later, total RNA was isolated for cDNA synthesis from 3 mL of culture wells. All qPCR reaction mixtures contained equal amount of cDNA from the treated or untreated cultures. **A** Chloroquine (CQ) treatment upregulated all genes, but *PbATG5* and *PbATG12* transcription levels were significantly higher than *PbATG8* and *PbMCA2*. **B** Tunicamycin (TN) treatment resulted in downregulation of *PbATG8* and *PbATG12*, and upregulation of *PbATG5* and *PbMCA2*. **C** 5-Fluorouracil (5-FU) treatments upregulated all genes similar to CQ treatment*.* Experiments were performed in three replicates. Results were shown as fold changes relative to the untreated control. The positive fold changes indicate the upregulation as a result of the drug treatments, and the negative fold changes indicate the downregulation. Each bar shows mean ± SD values for each gene. *p < 0.05, ***p < 0.001, and ****p < 0.0001

Prolonged effects of ER stress are known to induce autophagy and apoptosis pathways (Galluzzi et al. 2017; Kamil et al. 2017). Therefore, we further analyzed the transcription levels of autophagy-related genes (*Pb*ATG5, *Pb*ATG8, and *Pb*ATG12), and the metacaspase, *Pb*MCA2, gene under drug-induced ER stress conditions to see the effect of ER stress on common markers of PCD pathways. All four genes showed elevated levels of transcription under CQ (Fig. 3A) and 5-FU (Fig. 3B) treatments. In contrast, *ATG5* and *MCA2* were upregulated, but *ATG8* and *ATG12* were downregulated under TN treatment (Fig. 3C).

## Discussion

The life cycle of *Plasmodium* parasites involves two distinct host organisms, multiple tissues, various host cells, and very different environments. Parasites adapt to changing conditions by constantly altering their morphology and physiology, as well as altering their host cells. These alterations naturally require heavy trafficking of large repertoire of intracellular and secreted proteins that leads to heavy protein loads in ER, consequently triggering stress responses (Kaiser et al. 2016). Exploiting ER stress is a common strategy in drug development, but repurposing of existing drugs is also attracting attention. Therefore, it is important to understand parasitic stress responses and PCD pathways of parasitic protozoans in response to chemical compounds.

In this study, the effects of three ER stressor drugs, with various effector mechanisms, on the transcriptions of three autophagy genes and one metacaspase gene were investigated. Ex vivo* P. berghei* cultures were treated with the selected drugs: chloroquine, tunicamycin, and 5-fluorouracil.

Chloroquine is the most common quinoline derivate antimalarial which inhibits detoxification of hematin, a byproduct of host erythrocyte hemoglobin, causing high toxicity. In addition, quinoline derivatives have been postulated to have various antimalarial and anticancer roles such as inhibition of tyrosine kinase (Kaur et al. 2010) and inhibition of autophagosome-lysosome fusion (Mauthe et al. 2018; Kamil et al. 2022).

Tunicamycin is known for its ability to induce ER stress by inhibiting N-glycosylation of proteins, resulting in accumulation of unfolded proteins in the ER lumen. It has antiviral, antibiotic, antifungal, and antitumor activities (Myers et al. 1993), but is not suitable for clinical use due to its toxicity in mammalian cells.

5-Fluorouracil is used as an anticancer drug, and it was shown to induce high levels of ER stress (Yao et al. 2020). It also has an indirect role in negative drug selection in genetic manipulation of *Plasmodium* species due to its high toxicity (Manzoni et al. 2014). 5-FU is a uracil analogue that has a fluorine atom at its fifth carbon. It is transported and converted to fluorodeoxyuridine monophosphate (FdUMP) which binds and inhibits thymidine synthase, preventing the synthesis of deoxythymidine monophosphate (dTMP). Depletion of dTMP leads to severe disruption of DNA synthesis and repair (Longley et al. 2003). 5-FU might create more severe effects in *Plasmodium* species due to the extremely AT-rich genome structure.

Our results showed that treatments of parasite cultures with all three drugs induced significant levels of ER stress and altered transcription profiles of autophagy and apoptosis-related genes. Tunicamycin-induced ER stress usually leads to apoptosis (Guha et al. 2017). TN treatment upregulated *MCA2* as expected, but it also upregulated *ATG5* together with the downregulation of *ATG8* and *ATG12*. Atg5 had previously been shown to have pro-apoptotic roles in mammalian cells (Codogno and Meijer 2006). This expression profile suggests induction of an autophagy-induced apoptosis-like cell death mechanism. Same line of thinking might also be applied to expression profiles of CQ and 5-FU treatments; induction of all four genes could be explained by a similar autophagy-induced apoptosis-like pathway. Prolonged or excessive autophagy is known to lead to cell death. Other likely explanation is the excessive levels of ER stress could induce more than one response pathways simultaneously. Similarly, DNA damage can induce apoptosis, but it has also been shown to induce autophagy (Rodriguez-Rocha et al. 2011). Therefore, DNA replication and repair inhibitions by 5-FU treatments might lead to activation of both autophagy and apoptosis-like pathways.

CQ and 5-FU treatments induced significantly elevated levels of *BiP* upregulation, but as a noticeable difference, CQ treatment caused drastically upregulated transcription levels of *ATG5* and *ATG12* genes. Atg5 and Atg12 are required to form autophagopore, and Atg8 is required for the elongation and closure of autophagosome membrane (Hain and Bosch 2013). In previous studies, CQ treatment resulted with the cytosolic accumulation of autophagic vesicles, and CQ treatment was reported to inhibit autophagosome-lysosome fusion in an autophagy-independent manner (Mauthe et al. 2018). Thus, noticeable upregulation of the *ATG5* and *ATG12* transcripts in response to CQ treatment could be the result of autophagosome-lysosome fusion inhibition.

Although this is a limited study, our results indicate that *Plasmodium* parasites respond to various ER stressors by inducing autophagy- and/or apoptosis-like pathways. Atg8 is generally accepted as a classical autophagy marker, but as an important limitation of this study, there are no established markers for apoptosis in *Plasmodium*. More work is required to overcome this limitation and uncover PCD pathways of malaria parasites. Further studies are warranted to explore relationship between autophagic and apoptotic pathways, especially at the translational level, which is likely to provide interesting results.

## Data Availability

Data will be made publicly available upon publication and upon request for peer review.
