# Correction to: Drug‑induced ER stress leads to induction of programmed cell death pathways of the malaria parasite

**DOI:** 10.1007/s00436-024-08355-2

**Published:** 2024-10-05

**Authors:** Sinem Unal, Umit Y. Kina, Mohd Kamil, Ahmed S. I. Aly, Bedia Palabiyik

**Affiliations:** 1 Aly Lab, Beykoz Institute of Life Sciences and Biotechnology, Bezmialem Vakif University, Istanbul, 34820 Turkey; 2 Department of Molecular Biology and Genetics, Institute of Graduate Studies in Sciences, Istanbul, University, 34134 Istanbul, Turkey; 3 Center for Global Infectious Disease Research, Seattle Children’s Research Institute, 307, Westlake Ave N, Seattle, WA USA; 4 School of Science and Engineering, Al Akhawayn University, 53000 Ifrane, Morocco; 5 Faculty of Science, Department of Molecular Biology and Genetics, Istanbul University, 34134 Istanbul, Turkey

**Correction to: Parasitology Research (2024) 123:263**

10.1007/s00436-024-08281-3

The authors regret that the project number that appears in the funding section of the original published article is incorrect. The correct project number is 37560 instead of 34860.

The correct funding section appears below:

**Funding** Open access funding provided by the Scientific and Technological Research Council of Türkiye (TÜBİTAK). This work was supported by the Scientific Research Projects Coordination Unit of Istanbul University. Project number: 37560.

The original article has been corrected.

